# Expression of Toll-like Receptors and Chemokine Receptors in the Early and Advanced Stages of HIV Infection Aggravated by *Mycobacterium tuberculosis*

**DOI:** 10.3390/ijms27188161

**Published:** 2026-09-14

**Authors:** Marina Nosik, Elizaveta Bystritskaya, Konstantin Ryzhov, Elizaveta Kostyuchenko, Anna Kuzina, Alexey Kravtchenko, Sergei Sevostyanihin, Igor Demenok, Vitaly Zverev, Oxana Svitich

**Affiliations:** 1 I.I. Mechnikov Institute of Vaccines and Sera, 105064 Moscow, Russia; lisabystritskaya@gmail.com (E.B.); rkazaw@yahoo.com (K.R.); pontigruel@gmail.com (E.K.); kuzvitanna@yandex.ru (A.K.); vitalyzverev@outlook.com (V.Z.); svitichoa@yandex.ru (O.S.); 2 Department of Microbiology, Virology and Immunology, I.M. Sechenov First Moscow State Medical University, 119048 Moscow, Russia; 3 Central Research Institute of Epidemiology, Rospotrebnadzor, 111123 Moscow, Russia; alexey-kravtchenko@yandex.ru; 4 G.A. Zaharyan Moscow Tuberculosis Clinic, 125466 Moscow, Russia; sesevost@yandex.ru (S.S.); demenokin@zdrav.mos.ru (I.D.)

**Keywords:** HIV, *Mtb* infection, TLR, CCR, CXCR, IL-12, IL-18

## Abstract

This study examined Toll-like receptors (TLRs)/chemokine receptors (CCRs) gene expression levels in HIV-infected individuals at different stages of HIV infection. It has been demonstrated that in the advanced stages of HIV infection, aggravated by *Mycobacterium tuberculosis* (*M. tuberculosis*) infection, there was a significant increase in *TLR2*, *TLR4*, *TLR7*, and *TLR9* expression levels (181-, 8.4-, 15-, and 3.4-fold, respectively), compared to the early stages of the disease, which also correlated with an increase in viral load. The advanced stages of HIV infection were also characterized by an increased *CCR5* expression level (8.08-fold, *p* < 0.0001) on patients’ mononuclear cells, which was not observed in the early stages of the disease. A positive correlation was found between the enhanced levels of *TLR2*/*TLR4* and *CCR5* expression, which facilitates the entry of HIV-1 into the cell (r = 0.03564, *p* = 0.0423 for *TLR2/TLR4*; r = 0.3793, *p* = 0.0269 for *CCR5*). The advanced stages of HIV infection were characterized by a sharp increase in the expression levels of CXCR4 and CCR8 co-receptors which also play an important role in the entry of HIV-1 into the host cell (more than 537-fold, *p* = 0.0006, and 5000-fold, *p* = 0.004, respectively). At the advanced stages of HIV infection, a positive correlation was found between increased production of TLR and CCR and elevated expression of IL-12 and IL-18. These factors contribute to virus persistence and chronic immune activation observed in patients with opportunistic infections. Thus, the obtained data indicate that HIV-1 in the advanced stages of infection induces an increased TLRs and CCRs expression, which, together with enhanced viral production, leads to chronic inflammation and can contribute to tissue damage and exacerbate the disease’s progression.

## 1. Introduction

HIV infection is characterized by a disruption of the body’s immune response [1,2,3,4]. In addition to the depletion of the CD4+ T-cell pool and reductions in the number and function of innate immune cells (such as plasmacytoid and myeloid dendritic cells), the advanced stages of the disease are further complicated by the development of opportunistic infections and decreased innate and adaptive immune responses to pathogens [1,5,6]. One of the most common co-infections in people living with HIV is tuberculosis (TB), which accounts for 60–68% of all opportunistic infections [7]. Currently, co-infection with HIV and TB is a major global health concern [3,8]. In 2024, 82% of people diagnosed with TB were also diagnosed with HIV [7]. Patients with HIV/TB co-infection have an extremely high risk of mortality, and in people living with HIV, TB is the leading cause of death [7,8]. In 2024 alone, 150,000 people with HIV died of TB [7].

HIV and TB are considered syndemic diseases that act synergistically by altering the body’s immune response to both pathogens, which increases the severity of each infection. Specifically, *Mycobacterium tuberculosis* (*Mtb*) suppresses the processing and presentation of major histocompatibility complex (MHC) class II HIV antigens in dendritic cells, thereby facilitating HIV-1 infection [9]. In turn, HIV-1 exacerbates the course of TB by causing HIV-infected cells to migrate to the periphery of the granuloma and disrupting the oxidative process of mycobacterial phagosomes, leading to impaired elimination of mycobacteria in alveolar macrophages [9,10,11,12].

The initial stage of the immune response involves the recognition of foreign pathogen-associated molecular patterns (PAMPs), for which the body employs a variety of innate immune response molecules (proinflammatory cytokines, chemokines, and defensins), with Toll-like receptors (TLRs) playing a key role [13,14,15,16]. For example, it has been found that in HIV/TB co-infection, there is a significant increase in the regulation of anti-apoptotic proteins in macrophages via TLR2, which contributes to the survival of both pathogens [9,17]. Additionally, there is evidence that *Mtb* can reactivate latent HIV in T cells through the downstream effects of TLR2 [9].

Currently, there is some knowledge about the role of innate immunity in HIV mono-infection and TB mono-infection; however, information regarding the production of innate immunity molecules in HIV infection complicated by *Mtb* infection is still quite limited. Therefore, the aim of this study was to investigate the production of various TLRs and chemokine co-receptors (CCRs) in HIV/TB co-infection. This paper presents the results of a comparative study on *TLR2*, *TLR4*, *TLR7*, *TLR9*, *CCR5*, *CXCR4*, and *CCR8* gene expression on the peripheral blood mononuclear cells (PBMCs) during the early stages of HIV infection, as well as in the advanced stages complicated by *Mtb* infection. Additionally, considering that both HIV infection and TB are characterized by chronic inflammation—even with successful treatment—the results of a correlation analysis between the production of specific pro-inflammatory cytokines (IL-12, IL-18), which play an important role in chronic immune activation, and the expression of the studied receptors are also presented.

## 2. Results

When studying TLRs/CCRs gene expression, patients with HIV were divided into two groups: the first group (n = 36) included patients with the early stages of HIV infection (stages 1–2, WHO classification) and the second group (n = 39) included patients with the advanced stages of HIV infection with TB accession (stages 3–4, WHO classification). Clinical and demographic characteristics of patients are given in Table 1. As about 46% of patients with the advanced stages had chronic hepatitis C virus (HCV), we also analyzed TLR/CCR expression in the patient group with dual infection with HIV/TB and HCV+ (HIV+/TB+/HCV+) vs. the patient group with dual infection with HIV/TB without HCV− (HIV+/TB+/HCV−). Since no difference in expression levels of studied receptors between those two groups was detected, we did not divide them into subgroups.

**Figure 1:**
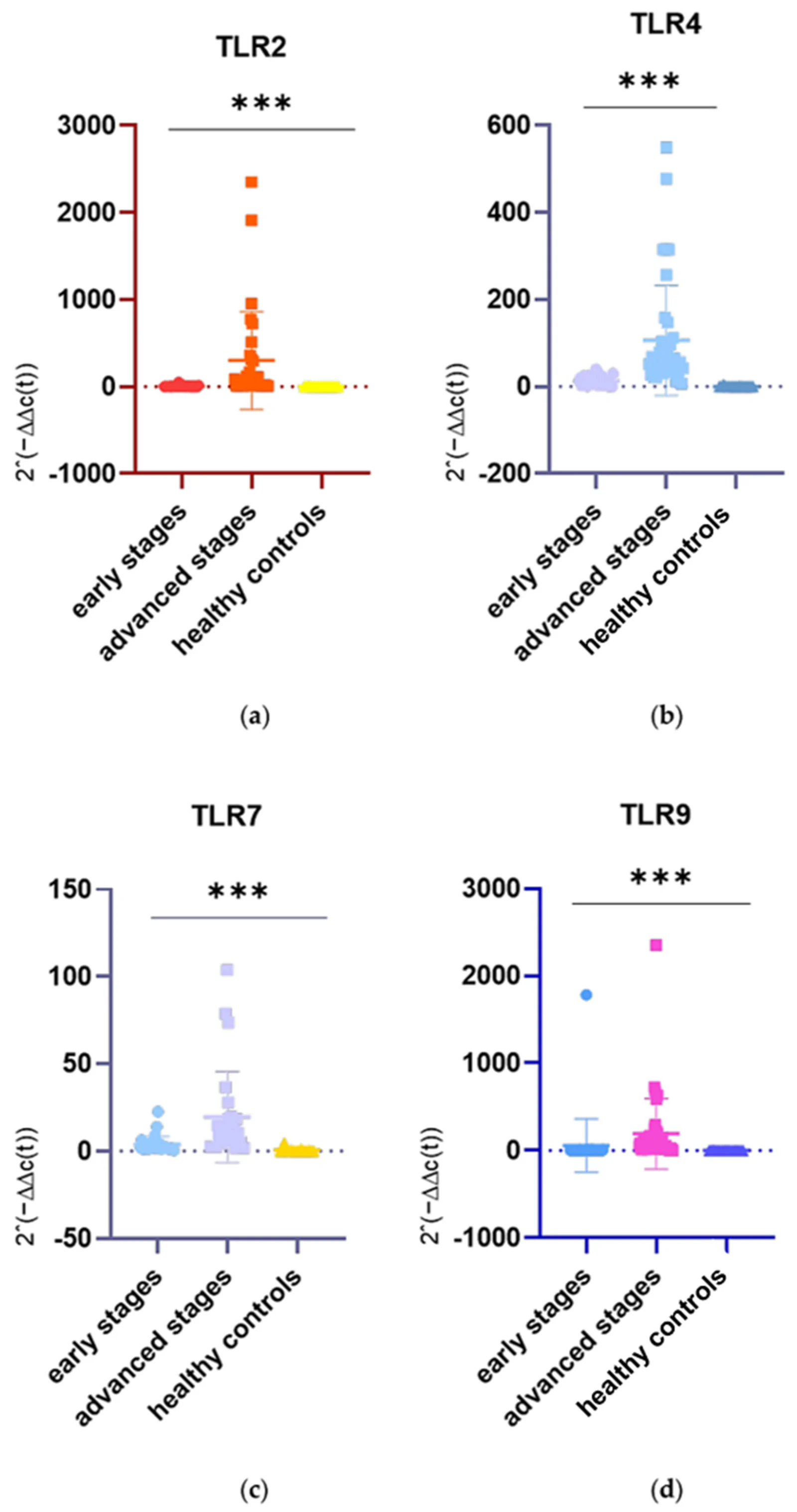
Median values of gene expression levels of TLRs on the PBMCs of patients with HIV at different stages of infection: (**a**) TLR2 expression. (**b**) TLR4 expression. (**c**) TLR7 expression. (**d**) TLR9 expression. 2^(−∆∆C(T))^, normalized expression coefficient. ***—*p*-value < 0.0001.

Patients in the second group were found to have opportunistic infections (OIs) such as tuberculosis (TB; 100%); chronic viral hepatitis C (HCV; 46.2%); cytomegalovirus infection (CMV; 12.8%) and candidiasis (10.3%). In the first group, HCV was detected in 13.9% of cases. The level of *TLR2* gene expression in patients with advanced HIV infection (group 2) was significantly increased (181-fold, *p* < 0.0001) compared to the group of patients with early stages (group 1) and the group of healthy controls (HCs), (more than 100-fold, *p* < 0.0001) (Figure 1a). However, there was no difference in *TLR2* RNA expression between patients in group 1 and HCs.

The level of *TLR4* expression in patients with early stages (group 1) of HIV infection was increased compared to the HCs group (128-fold, *p* < 0.0001). In patients with advanced HIV infection (group 2), the *TLR4* RNA expression level was 8.4 times higher than in patients from group 1 (*p* < 0.0001), and more than 1000 times higher than in HCs (*p* < 0.0001) (Figure 1b).

**Figure 2:**
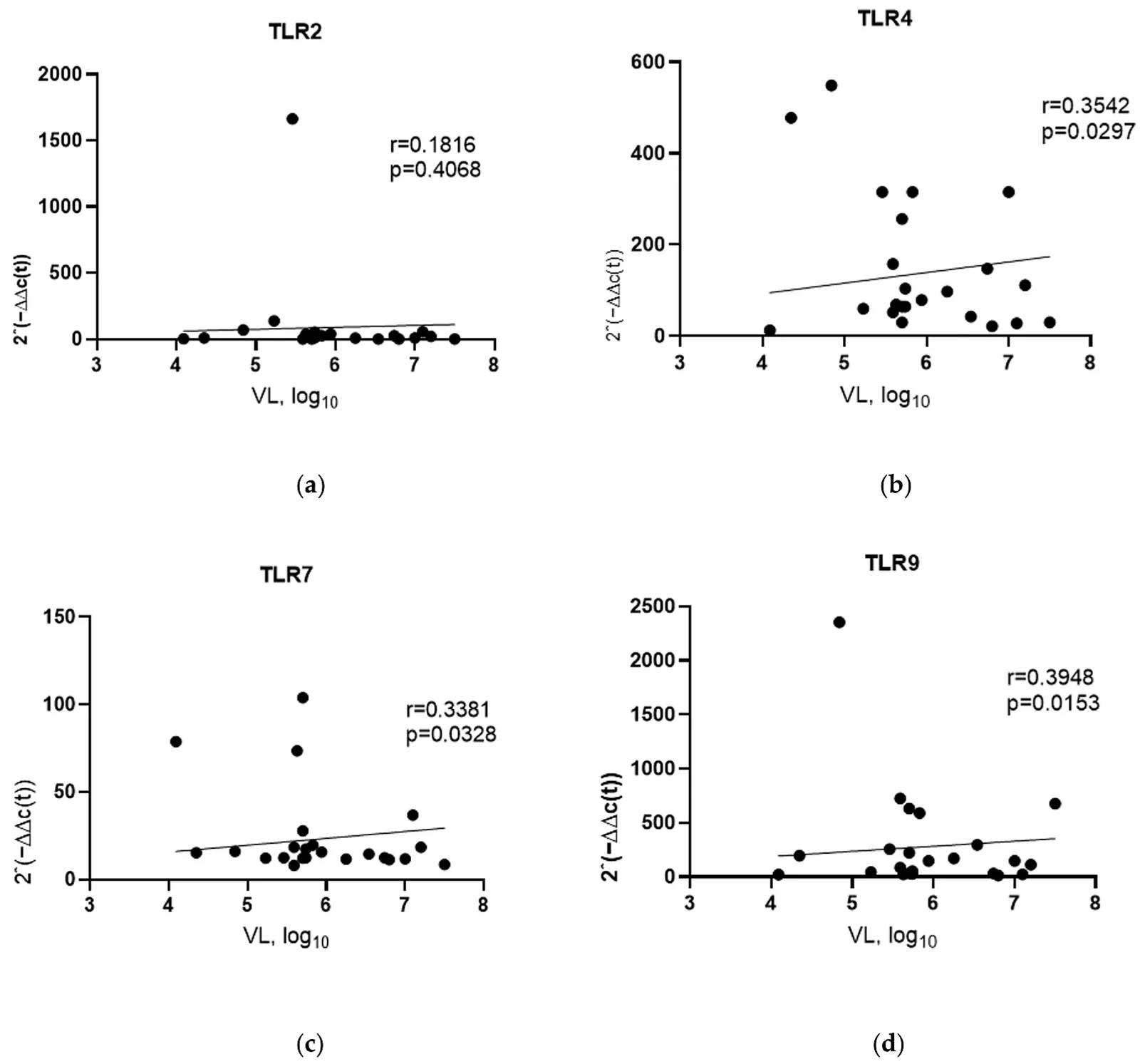
Correlation between the gene expression levels of TLRs and VL in patients with HIV in advanced stages of the disease: (**a**) TLR2. (**b**) TLR4. (**c**) TLR7. (**d**) TLR9. 2^(−∆∆C(T))^, normalized expression coefficient. VL, log10—viral load, copies/mL.

In patients with advanced stages of HIV infection (second group), a significant increase in the expression of *TLR7* and *TLR9* genes was observed, in comparison with the group of patients with initial stages (first group): for the *TLR7* gene 15-fold and that of the *TLR9* gene 3.4-fold, *p* < 0.0001, respectively, (Figure 1c,d). In patients with HIV from both groups, compared to the HCs group, *TLR7* gene expression was increased 22.2-fold in the first patient group and 41.7-fold in the second group, (*p* < 0.0001); *TLR9* expression level was increased more than 100-fold, (*p* < 0.0001). Increased gene expression of *TLR4*, -*7*, and *-9* in patients with advanced HIV infection correlated with an increased viral load (VL) (Figure 2). In the case of increased production of TLR2 in the TB group, there was no statistically significant correlation with VL (Figure 2a).

However, despite the expected difference in the number of CD4+ cells between the group of patients with early stages of HIV infection (median CD4+ = 541 cells) and advanced stages of infection (median CD4+ = 83 cells), there was no statistically significant correlation between TLR expression and the number of CD4+ cells in advanced stages of the disease (Supplementary Figure S1).

**Figure 3:**
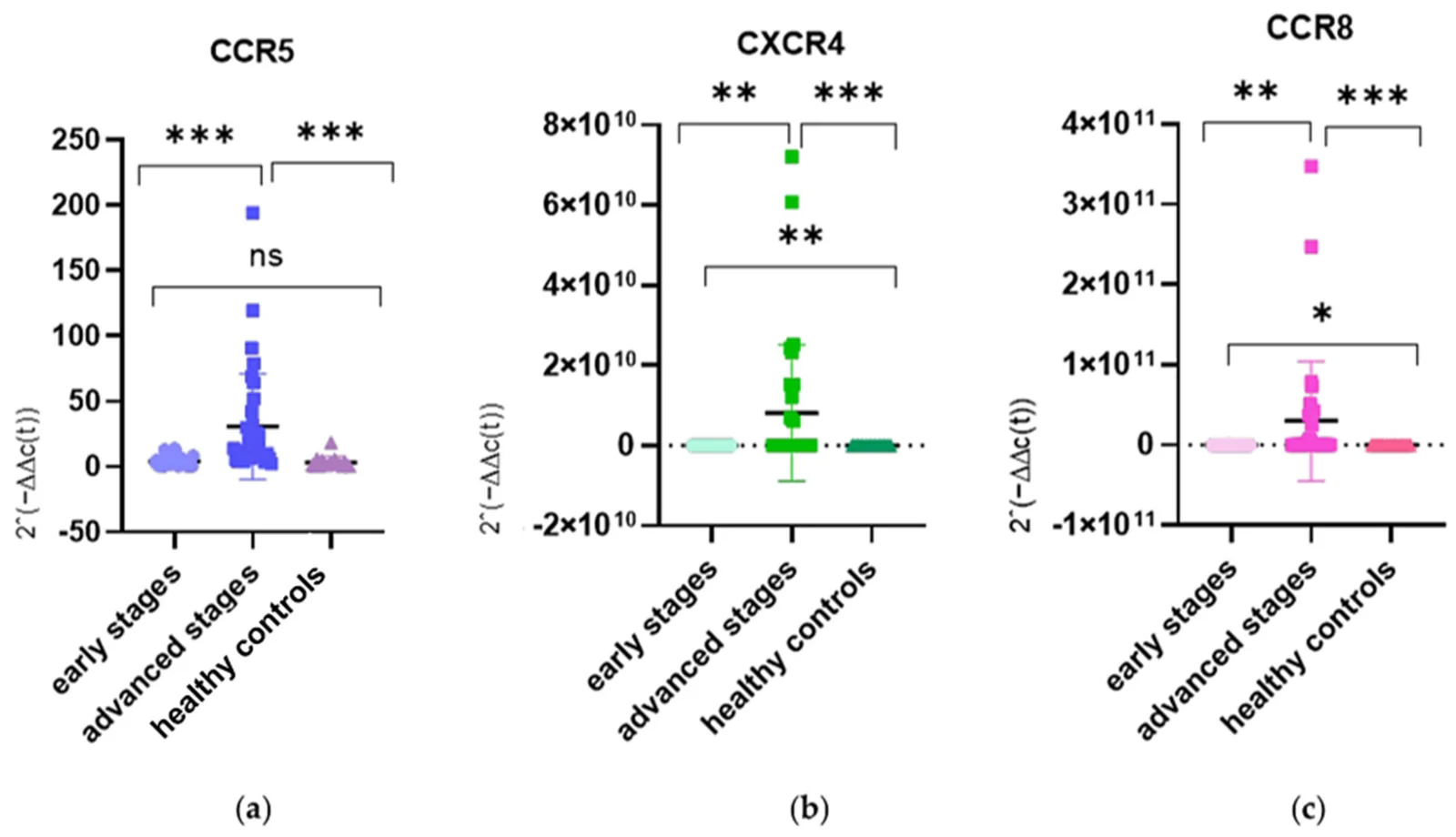
Median value of gene expression levels of chemokine co-receptors on the PBMCs of patients with HIV at different stages of infection: (**a**) CCR5 expression. (**b**) CXCR4 expression. (**c**) CCR8 expression. 2^(−∆∆C(T))^, normalized expression coefficient. ***—*p*-value < 0.0001. **—*p*-value < 0.01. *—*p*-value < 0.05.

The study of chemokine co-receptor expression on the PBMCs of patients revealed that patients with advanced HIV infection (group 2) had significantly higher levels of *CCR5*, *CXCR4*, and *CCR8* than patients with early HIV infection (group 1) and HCs. The gene expression of *CCR5* in patients with advanced disease was 8.08 times higher (*p* < 0.0001) than in patients with early disease and 9.6 times higher (*p* < 0.0001) than in HCs (Figure 3a). Yet, there was no statistical difference in *CCR5* gene expression levels between patients with early stages of HIV infection and HCs (Figure 3a).

**Figure 4:**
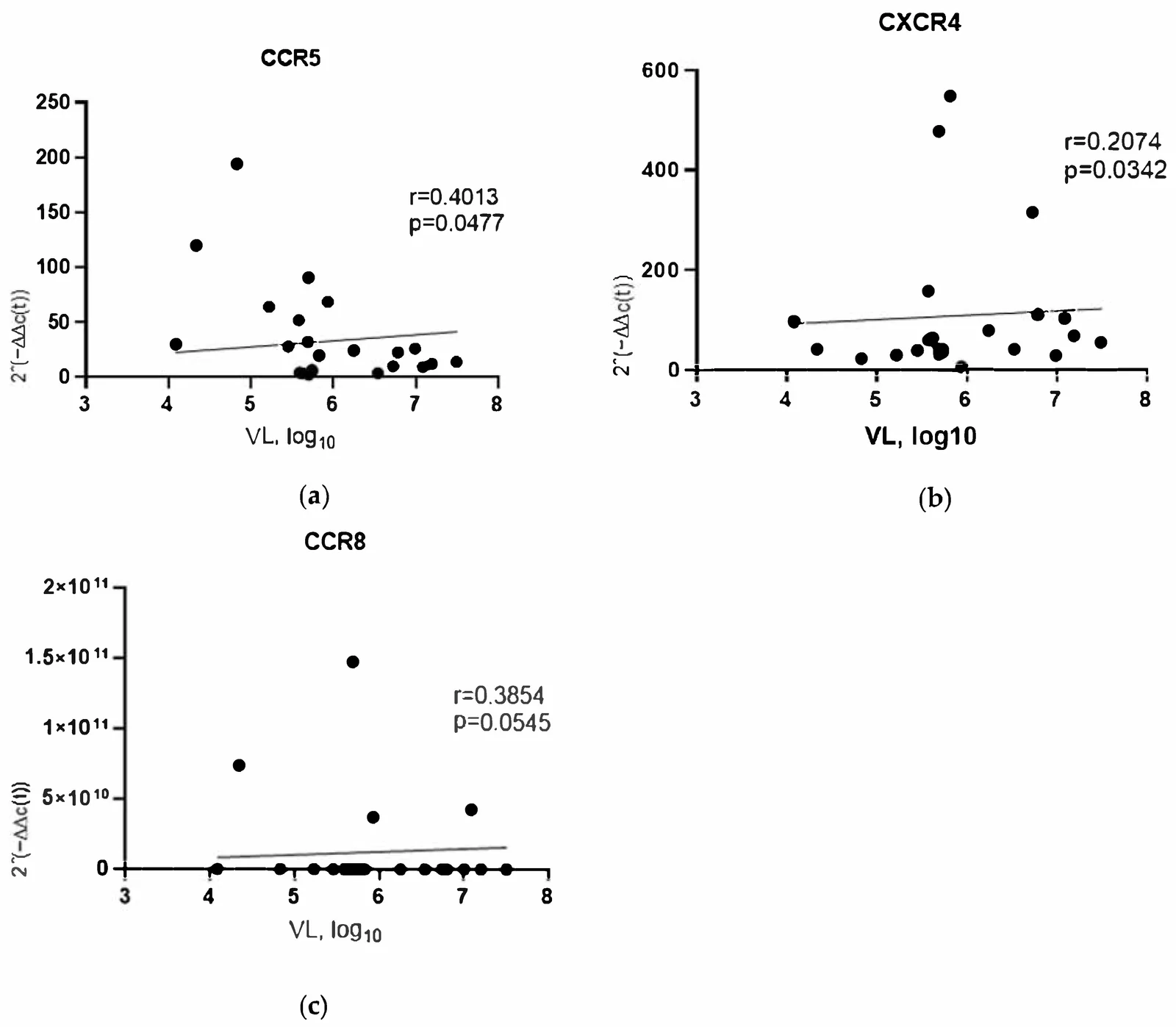
Correlation between the gene expression levels of chemokine receptors and VL in patients with HIV in advanced stages of the disease: (**a**) CCR5. (**b**) CXCR4. (**c**) CCR8. 2^(−∆∆C(T))^, normalized expression coefficient. VL, log10—viral load, copies/mL.

The expression levels of co-receptor genes *CXCR4* and *CCR8* were also significantly increased in the group of TB patients, compared with patients with the early stages: *CXCR4* more than 537-fold (*p* = 0.0006) and *CCR8* more than 5000-fold (*p* = 0.004), respectively; compared with HCs, the expression of both co-receptors was increased more than 100,000-fold (*p* < 0.0001) (Figure 3b,c). Increased expression of *CCR5* and *CXCR4* genes correlated with increased VL (*p* = 0.0477; *p* = 0.0342, respectively) (Figure 4a,b). In the case of CCR8, there was a tendency for correlation of enhanced co-receptor expression with viral replication (*p* = 0.0545) (Figure 4c).

**Figure 5:**
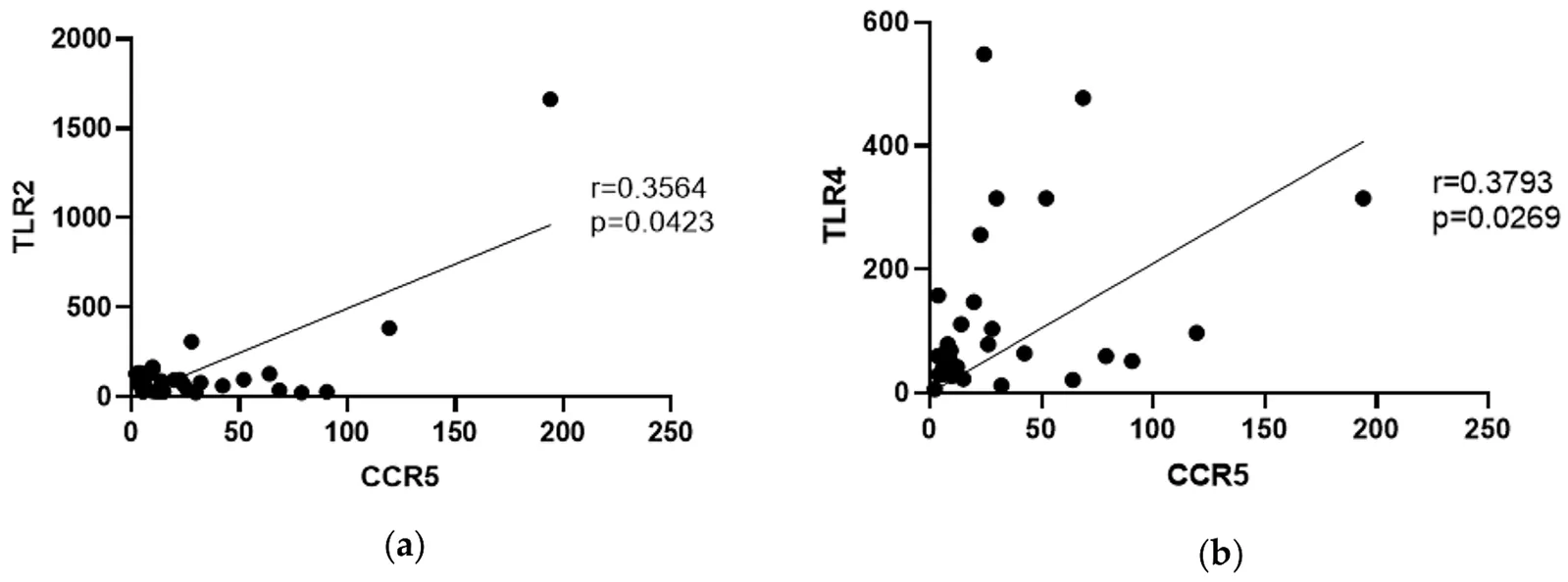
Correlation between the gene expression level of CCR5 and the production of Toll-like receptors in patients with HIV with advanced stages of the disease: (**a**) CCR5/TLR2. (**b**) CCR5/TLR4. 2^(−∆∆C(T))^, normalized expression coefficient.

In the advanced stages of HIV infection, increased production of *CCR5* was also correlated with increased expression levels of *TLR2* and *TLR4* genes: r = 0.3564, *p* = 0.0423 and r = 0.3793, *p* = 0.0269, respectively (Figure 5a,b). There was no statistically significant correlation between the expression of CXCR4/CCR8 co-receptors and the production of TLR2 and TLR4. There was also no correlation between the expression of co-receptors CCR5, CXCR4, and CCR8 and the expression of TLR7 and TLR9 (Supplementary Figure S2a,b).

**Figure 6:**
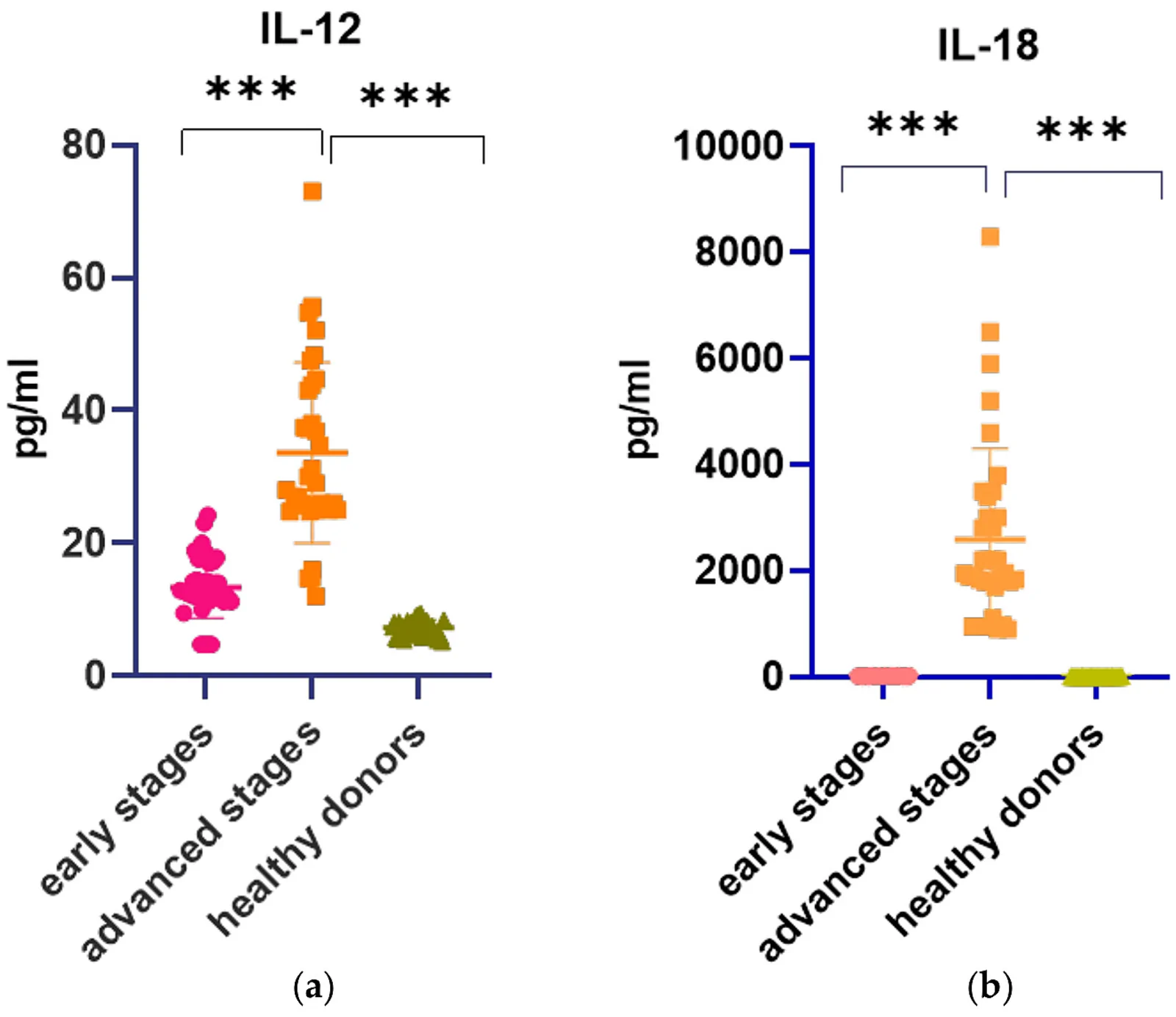
Median values of IL-12 and IL-18 production in patients with HIV in different stages of HIV infection: (**a**) IL-12 production. (**b**) IL-18 production. ***—*p*-value < 0.0001.

Since HIV infection is characterized by abnormal hyperimmune activation and inflammation, which persist to a certain extent even with antiretroviral therapy (ART), the relationship between the gene expression of TLR2, TLR4, TLR7, and TLR9 and the cytokines IL-12 and IL-18, whose persistent production leads to chronic immune activation, has been studied. The level of cytokine IL-12 production, which induces HIV-1 replication, was increased 1.8-fold in the group of patients with early stages of HIV infection compared to the HCs group (*p* < 0.0001) (Figure 6a). In the advanced-stage group, IL-12 production was dramatically increased compared to the early-stage group and the HCs group: a 193-fold increase, *p* < 0.0001, and more than a 300-fold increase, *p* < 0.0001, respectively (Figure 6a).

**Figure 7:**
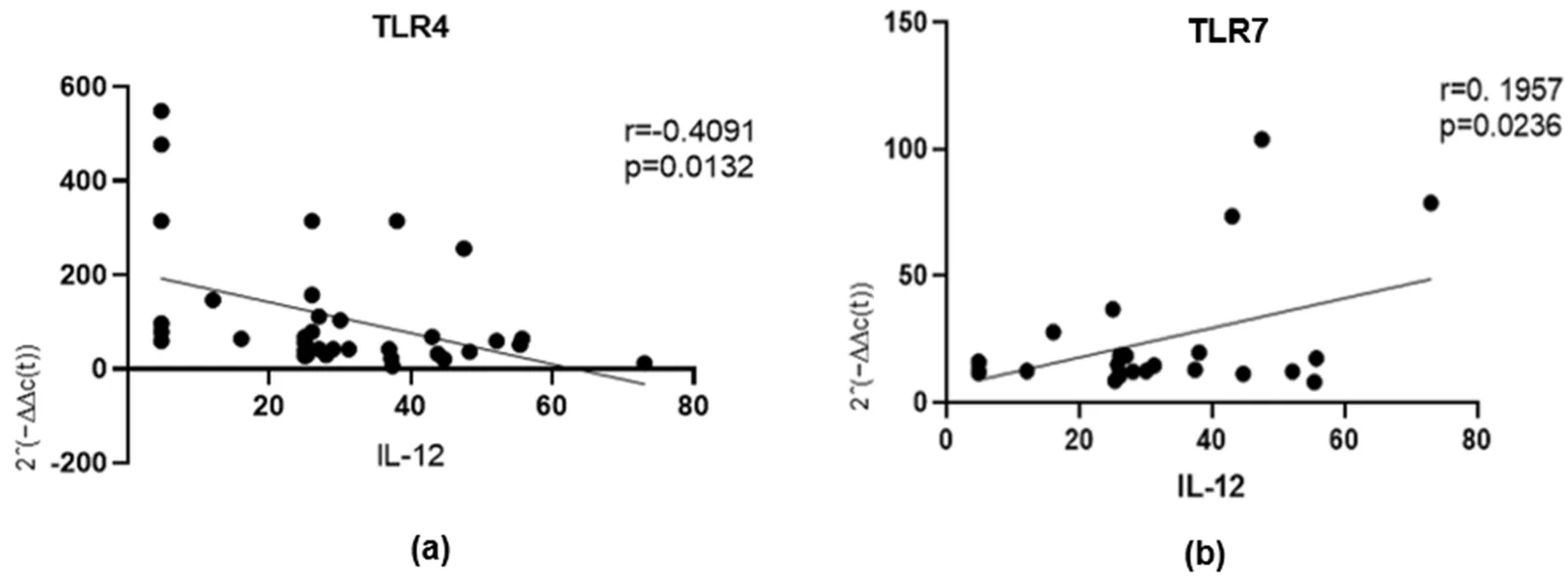
Correlation between TLR gene expression levels and IL-12 production in patients with HIV with advanced stages of HIV infection: (**a**) IL-12/TLR4. (**b**) IL-12/TLR7. 2^(−∆∆C(T))^, normalized expression coefficient.

At the same time, if in the early stages of HIV infection there was no correlation between the increased expression of any of the studied TLRs and a high level of IL-12 (Supplementary Figure S3a,b), then in the advanced stages of HIV infection there was a positive correlation between increased *TLR7* gene expression and increased IL-12 production (r = 0.1957, *p* = 0.0336), and a negative correlation with *TLR4* gene expression level (r = −0.4091, *p* = 0.0132) (Figure 7a,b).

**Figure 8:**
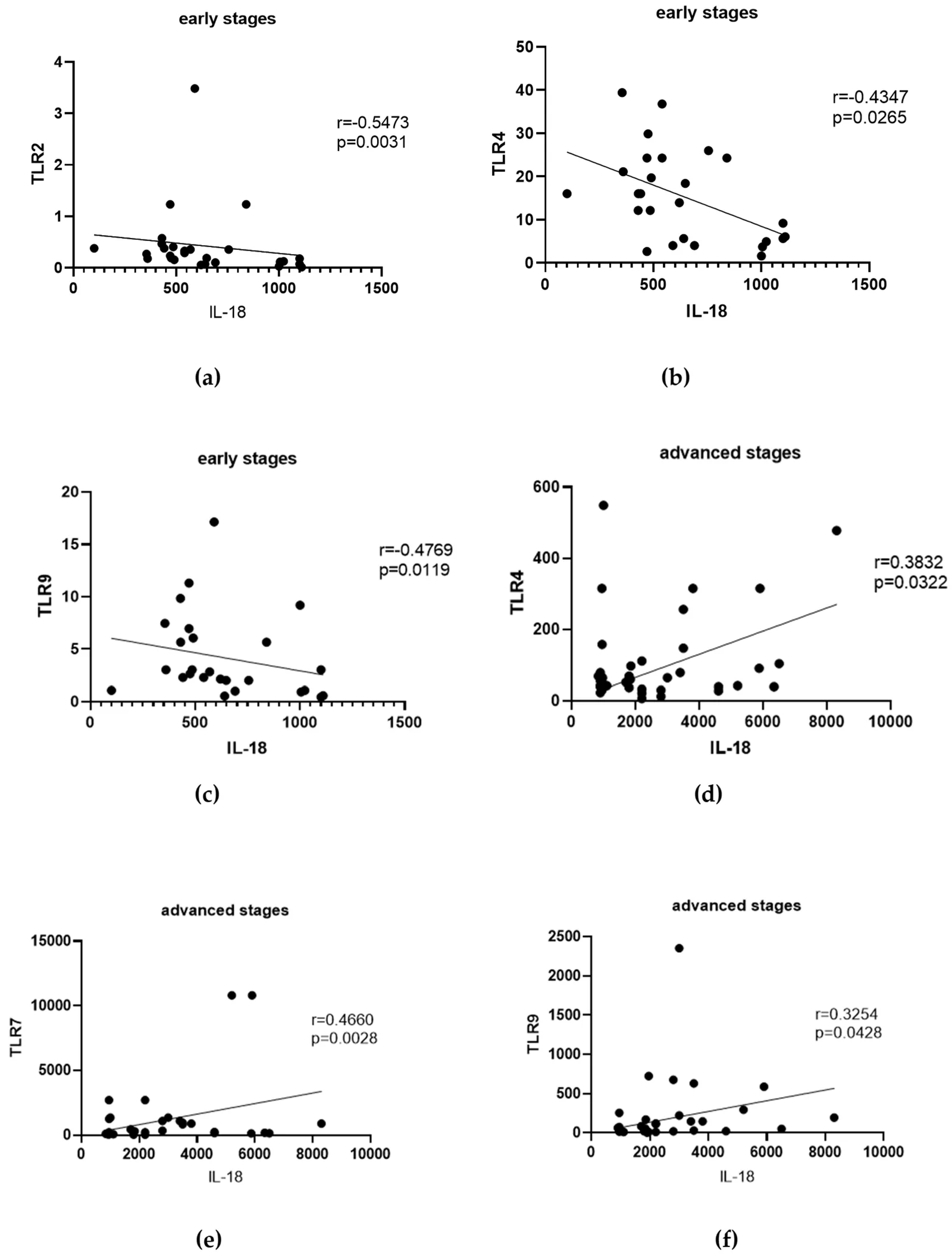
Correlation between the gene expression levels of TLRs and the production of IL-18 in patients with HIV at different stages of infection: (**a**) IL-18/TLR2, early stages. (**b**) IL-18/TLR4, early stages. (**c**) IL-18/TLR9, early stages. (**d**) IL-18/TLR4, advanced stages. (**e**) IL-18/TLR7, advanced stages. (**f**) IL-18/TLR9, advanced stages. 2^(−∆∆C(T))^, normalized expression coefficient.

The production of the HIV-1 replication-inducing cytokine IL-18 was increased 1.4-fold in the group of patients with early stages of HIV infection compared to the HCs group (*p* < 0.0001) (Figure 6b). In the TB group, IL-18 production was dramatically increased, almost 19-fold, compared to the HCs group (*p* < 0.0001) and 13-fold (*p* < 0.0001) compared to the group of patients with early stages of HIV infection (Figure 6b). In the early stages of the disease, a negative correlation was found between the production of this cytokine and the expression of *TLR2*, *TLR4*, and *TLR9*: r = −0.5473, *p* = 0.0031; r = −0.4347, *p* = 0.0265; and r = −0.4769, *p* = 0.0119, respectively (Figure 8a–c). In contrast, at the stages of HIV infection characterized by the accession of TB, a positive correlation was found between the production of IL-18 and the expression of *TLR4*, *TLR7*, and *TLR9*, with the exception of *TLR2*: r = 0.3832, *p* = 0.0322; r = 0.4660, *p* = 0.0028; and r = 0.3254, *p* = 0.0428, respectively (Figure 8d–f). No statistical correlation was found between IL-18 production and TLR7 expression levels at the early stages of the disease, as well as between TLR2 expression levels at the advanced stages (Supplementary Figure S4a,b).

**Figure 9:**
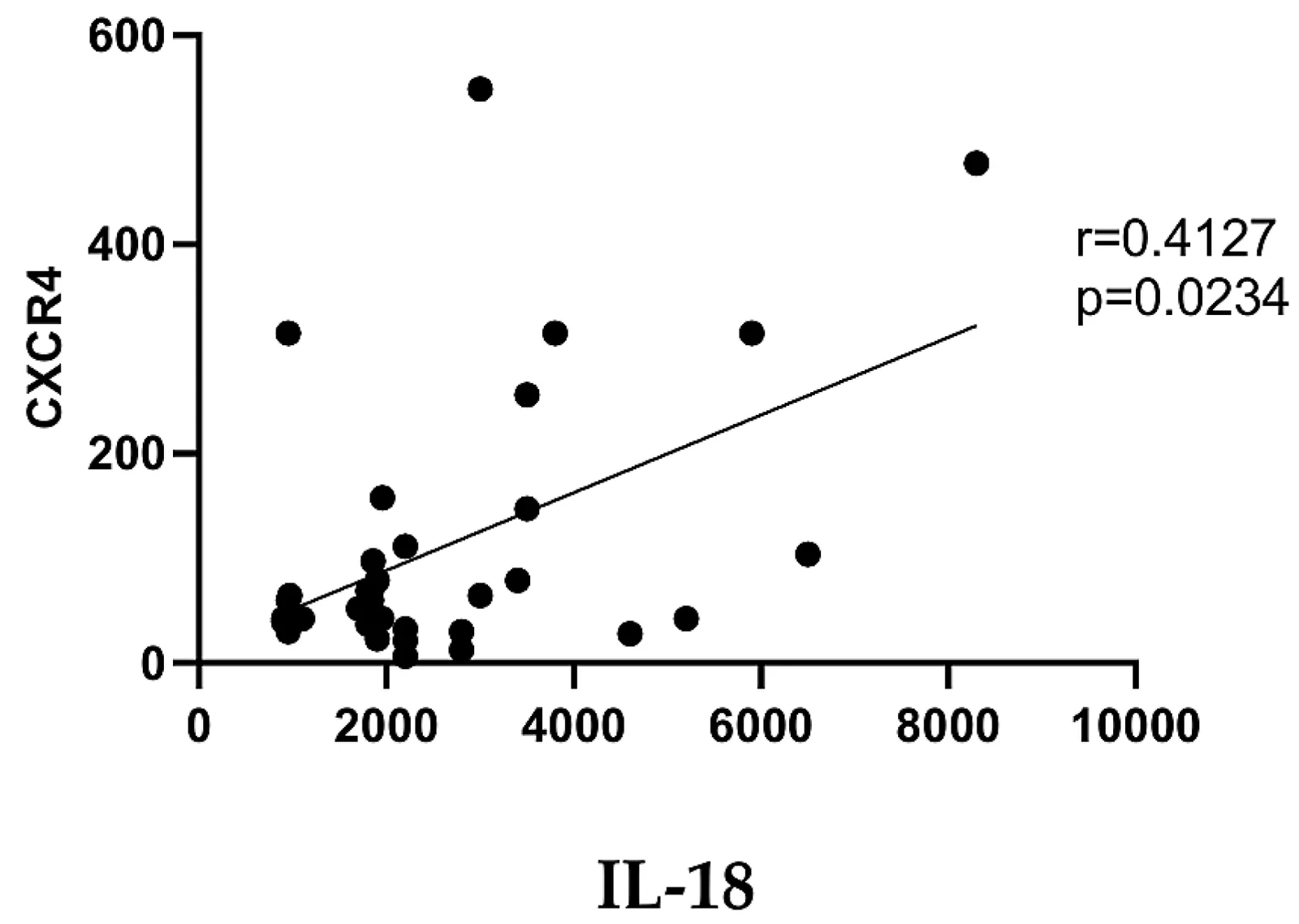
Correlation between the gene expression level of CXCR4 and the production of IL-18 in patients with HIV in advanced stages of infection. 2^(−∆∆C(T))^, normalized expression coefficient.

Also, a correlation was found between an increased gene expression of the *CXCR4* co-receptor and increased production of IL-18 in patients in advanced stages of HIV infection (r = 0.4127, *p* = 0.0234) (Figure 9). No statistically significant correlation was found between *CCR5* and *CCR8* gene expression and IL-18 production (Supplementary Figure S5a,b).

## 3. Discussion

The study results showed that HIV-1 in the advanced stages of HIV infection induces increased gene expression of *TLR2*, *TLR4*, *TLR7*, and *TLR9*, as well as chemokine co-receptors *CCR5*, *CXCR4*, and *CCR8*, which are involved in innate immune response reactions. This, along with the increased production of pro-inflammatory cytokines IL-12 and IL-18, leads to chronic inflammation and chronic immune activation, in addition to increased viral replication.

It is well known that TLRs are the main modulators of innate immunity. They play a key role in the immune defense of the body, determining the outcome of microbial and viral infections [13,18,19,20]. Recent studies have shown that TLRs play an important role in HIV infection [9,16,18,19,21]. TLRs directly interact with HIV-infected cells and induce the activation of signaling pathways that can regulate HIV-1 replication [19,21,22]. In the TLR-dependent immune response, cells that are not fully susceptible to HIV-1 infection can also participate, triggering a process of cell-mediated responses that affect viral production [19,22]. Moreover, defective viral particles that are unable to replicate fully and are presented in significantly higher quantities than infectious particles can still profoundly impact the activation of the TLR-dependent response in HIV infection [19,22,23]. The study revealed that HIV-1 infection promotes increased gene expression of *TLR4*, *TLR7*, and *TLR9*, regardless of the stage of the disease, compared to healthy donors. This is consistent with other studies that have shown increased expression of TLR4, TLR7, and TLR9 in HIV mono-infection [24,25,26]. The exception was TLR2, whose expression was comparable to that of healthy donors in patients at the early stages. Meanwhile, in the advanced stages of HIV infection complicated by TB, there was a significant increase in the gene expression of all four TLRs (*TLR2*, *TLR4*, *TLR7*, and *TLR9*) compared to the early stages. In particular, there was a sharp increase in TLR2, whose gene expression level was increased 181-fold (*p* < 0.0001).

Notably, as in the case of HIV infection, TLRs, along with other receptors, play a crucial role in the recognition of *Mtb*. For example, in TB mono-infection, there is an increased production of TLR2 and TLR4 [27]. TLR2 and TLR4 recognize *Mtb* through specific components of the mycobacterial cell wall, such as lipoarabinomannan, *Mtb* 19 kDa lipoprotein, lipomannan, and phosphatidyl-myo-inositol mannoside [27,28]. After recognition of these receptors, the TLR signaling pathway is activated by binding to the adaptor protein MyD88, followed by the activation of protein kinase, which leads to the activation of key transcription factors such as NF-κB [15,27,28,29,30]. The signaling activation of TLRs is essential for the formation of phagolysosomes and the elimination of the pathogen in infected alveolar macrophages, which are the first line of defense against *Mtb* [27,28,30]. Thus, a sharp increase in the gene expression of *TLR2* and *TLR4* in patients with HIV with TB infection, can be explained by the fact that TLR2 and TLR4 are important detectors of *Mtb*. In addition, the results of some studies suggest that a high level of TLR2 plays a particularly important protective role in chronic *Mtb* infection [28,31]. TLR7 and TLR9 are also *Mtb* infection detectors, recognizing unmethylated CpG regions of mycobacterial DNA, followed by recruitment of the adaptor protein MyD88, translocation of activated NF-κB, and induction of pro-inflammatory cytokine production [9,13,15,32]. It has been shown that TLR7 and TLR9 are capable of inducing MHC class II expression via the activation of mitogen-activated protein kinase (MAPK) and AP-1/CREB, thereby increasing the amount of MHC class II for optimal interaction with CD4+ cells and their activation to protect against *Mtb* [9,14]. Thus, the increased gene expression of *TLR7* and *TLR9* in patients with advanced HIV infection compared to patients with early HIV infection, as in the case of TLR2 and TLR4, is explained by the body’s protective immune response against *Mtb* invasion. However, increased gene expression levels of *TLR2*, *TLR4*, *TLR7*, and *TLR9* have a dual effect. While increased expression of these TLRs plays a protective role in TB, it has the opposite effect in HIV infection. Numerous studies have shown that HIV-1 induces changes in TLR expression, leading to increased production of HIV-1 [19,21,33,34,35]. Several studies have shown that increased TLR2 expression can be associated with enhanced viral replication [15,24,33]. Direct binding of HIV-1 structural proteins (p17, p24, and gp41) to TLR2 can significantly enhance cellular activation and increase the amount of proviral DNA [15]. It has also been demonstrated that increased expression of TLR4 is associated with the progression of HIV infection [24,25,26]. The HIV-1 structural protein gp120 can directly bind to TLR4, modulating HIV-1 replication [36]. There is evidence that the presence of certain single nucleotide polymorphisms (SNPs) in the *TLR4* gene, which are most commonly found in individuals with HIV, correlates with increased VL [37,38,39]. Increased gene expression of *TLR7* and *TLR9* in HIV infection also has negative consequences. Persistent TLR7 increased production indirectly induces apoptosis in T cells, which in turn leads to unintentional apoptosis of uninfected CD4+ T cells and depletion of the CD4+ T-cell pool [9]. In addition, TLR7-induced T-cell anergy helps HIV-1 avoid elimination, promoting persistent viral replication [9,19,40]. TLR9 expression is also associated with increased concentrations of HIV-1 RNA [34]. There is evidence that chronic activation of TLR9, caused by co-infection with *Mtb*, induces increased production of pro-inflammatory cytokines, which, by binding to the long terminal repeat (LTR) of HIV-1, activates HIV-1 transcription [9]. These data are also supported by the results of this study, which revealed a correlation between increased gene expression of *TLR4*, *TLR7*, and *TLR9* and increased VL in the advanced stages of HIV infection. However, as mentioned earlier, TLR2 was an exception. In our study, despite the significantly increased gene expression level of *TLR2* in patients with advanced stages of the disease, there was no statistically significant correlation with VL, which is consistent with the findings of several other studies [14,26]. This may be partially explained by the presence of SNPs, as some studies have shown that susceptibility to HIV-1 infection is associated with the presence of a specific SNP in the *TLR2* gene [16,41,42].

TLRs control the immune response, which includes the production of chemokine co-receptors. CCR5 and CXCR4 are the main co-receptors used by HIV-1 to enter cells [43,44,45,46]. About 46% of HIV-1 isolates can use both co-receptors simultaneously [47,48]. However, HIV-1 can also use alternative chemokine co-receptors to infect host cells [49,50]. It has been shown that more than 56% of HIV-1 isolates can use the CCR8, which is expressed not only on Th2 cells but also on monocytes, thymocytes, and phagocytes [51,52,53,54,55,56]. The results of this study demonstrated a significant increase in the expression level of all three co-receptors (*CCR5*, *CXCR4*, and *CCR8*) in patients with advanced HIV infection, which is consistent with several other studies [15,57,58,59]. However, while an increase in *CXCR4* and *CCR8* gene expression was observed in the early stages of infection compared to healthy donors, there was no statistically significant difference in the expression of the *CCR5* between early-stage patients and healthy donors. A significant increase in *CCR5* expression was observed in patients with advanced stages of HIV infection, compared to both healthy donors (9.6-fold increase, *p* < 0.0001) and patients with early stages of HIV infection (8.08-fold increase, *p* < 0.0001). This fact can be explained by the attachment of an *Mtb* infection. Increased gene expression level of *CCR5* in the advanced stages was correlated with increased expression of *TLR2* and *TLR4* (r = 0.03564, *p* = 0.0423 and r = 0.3793, *p* = 0.0269, respectively), which are responsible for inducing the production of this co-receptor [24,25]. In turn, increased expression of TLR2 and TLR4 is observed in TB [27,28].

While CCR5 is expressed on both T cells and macrophages, the CXCR4 is predominantly expressed on T cells [60]. However, it has been shown that if T cells encounter mycobacteria (both *Mtb* and non-tuberculous mycobacteria) in addition to HIV-1, they may be stimulated by certain mycobacterial cell wall products, leading to increased CXCR4 expression on macrophages [61]. Thus, with the onset of *Mtb* infection, the number of cells permissive to HIV infection increases, which indirectly leads to an increase in the reproductive activity of the virus. This fact may explain the sharp increase in CXCR4 expression (>537-fold, *p* = 0.0006) in advanced stages of the disease, which was identified in this study. The observed increased expression of *CCR8* (>5000-fold, *p* = 0.004) in the advanced stages of HIV infection with TB assessment may also be explained by the impact of *Mtb*. The role of CCR8 in TB is not fully understood, but it is possible that, as with CCR5 and CXCR4, infection with *Mtb* may increase the density of the CCR8 co-receptor on the surface of CD4+ *Mtb* antigen-specific T cells [10,62]. Given that CCR8 is involved in the formation of granulomas, it can be assumed that in the case of *Mtb* infection, especially within the granuloma, HIV-1 encounters a population of activated *Mtb* antigen-specific T cells that highly express this membrane receptor, which is necessary for HIV-1 infection [10,56]. Thus, the elevated expression levels of *CCR5*, *CXCR4*, and *CCR8*, which increase the permissiveness of cells to viral infection, can explain the increased VL characteristic of advanced stages of HIV infection.

It is known that both HIV infection, even with successful treatment, and TB are characterized by chronic immune activation [4,6,63,64,65,66,67,68,69,70,71]. HIV, due to its complex interactions with the body’s immune system, disrupts the immune response, leading to abnormal immune activation and chronic inflammation, which contributes to the rapid progression of the disease [64,66,68]. In turn, active TB is characterized by increased expression of pro-inflammatory and regulatory cytokines, which indicates increased immune activation and persistence of *Mtb* [4,63,72,73]. Chronic immune activation and inflammation observed in dual HIV/TB infection negatively affect the course of both diseases and lead to the development of complications such as cardiovascular diseases, neurocognitive diseases, cancer, osteoporosis, diabetes, and liver and kidney diseases [64,66,74,75,76]. Considering these facts, we studied the possible relationship between the expression of TLRs and CCRs in dual HIV/TB infection and the production of pro-inflammatory cytokines IL-12 and IL-18. It has been shown that HIV infection is characterized by dysregulation of cytokine expression and, in particular, increased expression of pro-inflammatory cytokines IL-12/IL-18, which indicates a state of chronic activation [4,77,78,79,80,81,82,83]. In this study, it was also revealed that the expression level of IL-12, which induces HIV-1 replication, was sharply increased (193-fold, *p* < 0.0001) in the group of patients with advanced stages via increased activation of CD4+ T cells, which are the main targets for HIV-1 [84,85]. At the same time, increased production of IL-12 in the advanced stages of HIV infection correlated with increased *TLR7* gene expression level. Given that increased expression of *TLR7* also contributes to chronic immune activation and is associated with increased VL [9,15], this fact explains the hyperimmune activation and viral persistence observed in advanced stages of HIV infection. Similarly, in the group of patients with *Mtb* infection, the production of the cytokine IL-18 was increased (13-fold, *p* < 0.0001), which, like IL-12, induces HIV-1 replication and is associated with abnormal immune activation [81,86,87]. At the same time, while in the early stages of the disease there was a negative correlation between IL-18 production and the expression of several TLRs, in the advanced stages, there was a positive correlation between increased IL-18 production and increased gene expression of *TLR4*, *-7*, and *-9*. Numerous data indicate that TLR4 plays an important role in the chronic activation of the immune system in HIV-1 infection [9,24,88]. It is suggested that in HIV infection, TLR4 expression contributes to increased immune activation by enhancing the immunosuppressive mechanisms of human plasmacytoid dendritic cells (pDCs) [89]. TLR9 is also responsible for the overactivation of the body’s immune response caused by HIV-1 infection [9,15]. Moreover, it has been shown that co-stimulation by TLR4 and TLR9 ligands, in addition to activation of the HIV-1 LTR and increased viral replication, leads to a mutually reinforcing production of pro-inflammatory cytokines [90]. In addition, a serious imbalance in the lymphocyte population caused by a long-term persisting HIV-1 infection can increase the risk of developing autoimmune processes in the advanced stages of HIV infection. There is evidence that in various autoimmune diseases, the interaction between the chemokine CXCL12 and its receptor CXCR4 leads to an increase in the number of M1 macrophages, which in turn stimulates the production of pro-inflammatory cytokines, including IL-18 [91]. Presumably, this fact may explain the identified positive correlation of IL-18 expression with increased CXCR4 production. Thus, the enhanced expression of IL-18 and *TLR4, -7,* and -*9* in the advanced stages of HIV infection together may lead to chronic immune activation and contribute to the virus persistence.

The negative correlation between IL-18 and the expression of several TLRs in the early stages of HIV infection may be explained by the fact that TLR signaling pathways are characterized by the presence of so-called negative regulators, which can interfere with ligand binding to the receptor, disrupting the protein–target interaction and inhibiting the production of intermediate products [92]. Soluble forms of TLRs (in particular, sTLR2 and sTLR4) can serve as feedback mechanisms for inhibiting excessive activation of TLRs and thereby suppressing the production of pro-inflammatory cytokines in the early stages of HIV infection [92,93,94,95,96].

## 4. Materials and Methods

### 4.1. Study Population

Patients with HIV infection (n = 75) were recruited from two large Moscow Medical Centers. HIV-seropositive status was confirmed using ELISA followed by Western blotting, in accordance with national regulations. TB diagnoses were based on clinical symptoms, sputum microscopy, and radiological analyses. Healthy controls (n = 40) were recruited from the general population at a blood transfusion center. Healthy controls (HCs) were repeatedly tested negative for HIV-1 and had no history of TB or exposure to the disease within the past 6 months. At the time of enrollment patients were naive to ART and anti-TB treatment.

### 4.2. Ethical Statement

All individuals enrolled in the study were over 18 years old and gave written informed consent for participation in the study. In accordance with the General Data Protection Regulation (GDPR), all participants were de-identified and anonymized by assigning them a unique code expressed as an identifier. All clinical samples, data, and study results were stored in anonymized form. The study was conducted following the guidelines of the Declaration of Helsinki and was approved by the Biomedical Ethics Committee of the I.I. Mechnikov Institute of Vaccines and Sera, Moscow, Russia.

### 4.3. Analysis of TLR and CCR Gene Expression Levels

PBMCs of individuals with HIV were isolated. Then, RNA was extracted from the PBMCs of donors using the ExtractRNA reagent, as per manufacturer’s instructions (RT-1 kit, Syntol, Moscow, Russia). The extracted RNA was used to obtain complementary DNA (cDNA) via a reverse transcription reaction. The obtained cDNA was used to determine the expression level of selected TLRs and CCRs using real-time polymerase chain reaction (RT-PCR). The Primer-BLAST program (version 2.5.0) (NCBI, Bethesda, MD, USA) was used to select specific sequences for primers synthesized by Syntol (Syntol, Moscow, Russia) that were complementary to the sites of the studied genes. The specific primer sets used were as follows: for *TLR2*, CCA-GCA-AAT-TAC-CTG-TGT-GA (forward primer) and CCC-ACA-TCA-TTT-TCA-TAT-AC (reverse primer); for *TLR4*, TTC-CCT-GGT-GAG-TGT-GAC-TA (forward primer) and CAC-CTT-TGT-TGG-AAG-TGA-AA (reverse primer); for *TLR7*, AAG-CCC-TTT-CAG-AAG-TCC-AAG-TT (forward primer) and GGT-GAG-CTT-GCG-GGT-TTG-T (reverse primer); for *TLR9*, TGG-TGT-TGA-AGG-ACA-GTT-CTC-TC (forward primer) and CAC-TCG-GAG-GTT-TCC-CAG-C (reverse primer); for *CCR5*, CTG-GCC-ATC-TCT-GACCTG-TTT-TTC (forward primer) and CAG-CCC-TGT-GCC-TCT-TCT-TCT-CAT (reverse primer); for *CXCR4* CTC-CTC-TTT-GTC-ATC-ACG-CTT-CC (forward primer) and GGA-TGA-GGA-CAC-TGC-TGT-AGA-G (reverse primer); for *CCR8*, TGG-CTG-TTG-TCC-ATG-CCG-TGT-A (forward primer) and TGG-GAT-GGT-AGC-CAT-AAT-GGC-G (reverse primer). The reaction was conducted under the following conditions: 1 cycle at 95 °C for 5 min and 40 cycles at 95 °C for 15 s and 60 °C for 50 s. Beta-actin was used as the reference gene for the analysis of TLR and CCR genes. The $2^{-∆∆C_{T}}$ method was used for the analysis of the relative changes in gene expression. It must be noted that the $2^{-∆∆C_{T}}$ transformation is exponential, and very low expression of a target gene in the healthy control group can generate numerically large fold changes even when the corresponding difference is less pronounced on the Ct scale. Therefore, these values represent relative ratios to a low control baseline and should not be interpreted as measures of absolute transcript abundance.

### 4.4. Monitoring of Viral Replication

The quantification of HIV-1 RNA in blood plasma was performed via a real-time reverse transcription–polymerase chain reaction (RT-PCR) using the “AmpliSens HIV Monitor-FRT” reagent kit (FSBI CRIE, Moscow, Russia). The study was performed on the real-time PCR cycler Rotor-Gene Q (Qiagen, Hilden, Germany) using standardized technology with automated sample preparation. The analysis of the results was carried out using the equipment software.

### 4.5. Assessment of Cytokine Expression

The plasma levels of the cytokine IL-18 were measured using the ELISA EIA-BEST Kit (Vector-Best, Moscow, Russia). The concentration of the cytokine IL-12 in blood plasma was determined using the IL-12 p70 ELISA kit (Servicebio, Wuhan, China) per the manufacturer’s recommendations. Plasma was isolated according to the standard procedure. The whole blood was collected in a vacutainer with EDTA and centrifuged at 1000 rpm for 15–20 min with cooling. Then, plasma was collected, aliquoted, and stored at −80 °C until further analysis. The cytokine concentrations were determined using a standard curve obtained with the standards provided by the manufacturer with each kit, and the results were expressed as pg/mL.

### 4.6. Statistical Analysis

The nonparametric Mann–Whitney U-test was used to compare two independent groups. The ANOVA test was used to compare three more independent groups. Dunn’s multiple comparison test was performed to determine the significant differences between the groups. The correlation was evaluated using Spearman’s rank correlation test. The data were analyzed using GraphPad Prism v9.5.0 (GraphPad Software, Boston, MA, USA). A *p*-value of < 0.05 (*) was considered significant; *p* < 0.01 (**) and *p* < 0.0001 (***) were considered very significant.

## 5. Conclusions

In summary, the study revealed that advanced stages of HIV infection complicated by TB are characterized by a sharp increase in gene expression levels of *TLR2*, *TLR4*, *TLR7*, and *TLR9*, which leads to enhanced replication of HIV-1. It has also been demonstrated that the joining of *Mtb* infection induces increased gene expression of *CCR5*, *CXCR4*, and *CCR8*, which play an important role in the entry of HIV-1 into the host cell, thereby enhancing viral replication activity. The increased production of CCRs in advanced stages of HIV infection, accompanied by increased expression of TLRs, explains the increased VL observed in advanced stages of HIV infection. Additionally, the increased production of TLRs and CCRs in the advanced stages of HIV infection, which correlates with increased production of pro-inflammatory cytokines such as IL-12 and IL-18, contributes to the persistence of the virus and the chronic immune activation observed in patients with advanced HIV infection and *Mtb* infection. Thus, in the advanced stages of HIV infection, HIV-1 induces overexpression of a number of TLRs and CCRs involved in the innate immune response, which, in addition to increased viral replication, leads to chronic inflammation and chronic immune activation, eventually resulting in immune system exhaustion and dysfunction, exacerbating the course of the disease. This study has some limitations, as we did not have the opportunity to conduct a comparative study of receptor expression in patients with HIV infection who were already receiving ART, which would have provided a more comprehensive understanding of the specific expression of TLRs/CCRs in dual HIV/TB infection. Also, this work has another limitation. In the frame of this study, we did not include data on TLRs and CCRs expression in patient group with TB mono-infection due to the small number of patients. However, based on numerous clinical and research data, it could be said with certainty that synergistic effect of both infections leads to hyperimmune activation. The results obtained provide important insights useful for future research in treating patients with HIV/TB co-infection. First, this involves the use of antagonists of various TLRs with the goal of reducing VL and suppressing hyperimmune activation. Given the complex and contradictory nature of the interplay between HIV-1, *Mtb*, and TLRs, which induces the production of pro-inflammatory cytokines and affects = VL, further detailed study of the role of individual TLRs in the pathogenesis of HIV infection complicated by TB is necessary.

## Figures and Tables

**Table 1:** Baseline characteristics of patients with HIV.

Characteristics	Early Stages (n = 36)	Advanced Stages (n = 39)
Gender, (n, %)		
Male	26/72.2	21/53.8
Female	10/27.8	18/46.2
Age (years), IQR		
Male	36 (23–54)	43.5 (29–57)
Female	33.8 (23–43)	44 (26–51)
CD4+ cells, IQR	684 (212–960)	155 (1–272)
Viral load (log10 copies/mL), IQR	4.53 (2.76–5.8)	6.7 (4.09–7)
Opportunistic infections (n, %)		
HCV	5/13.9	18/46.2
TB	−	39/100
CMV	−	5/12.8
Candidiasis	−	4/10.3

HCV—chronic hepatitis C virus; TB—tuberculosis; CMV—cytomegalovirus.

## Data Availability

The original contributions presented in this study are included in the article/Supplementary Materials. Further inquiries can be directed to the corresponding author.

## Supplementary Materials

The following supporting information can be downloaded at https://www.mdpi.com/article/10.3390/ijms27188161/s1.

## Abbreviations

The following abbreviations are used in this manuscript:

ART	Antiretroviral therapy
CCR	Chemokine receptor (C-C motif)
cDNA	Complementary DNA
CMV	Cytomegalovirus
CXCR	Chemokine receptor (C-X-C motif)
GDPR	General Data Protection Regulation
HCV	Chronic hepatitis C virus
HCs	Healthy controls
LTR	Long terminal repeat
MAPK	Mitogen-activated protein kinase
MHC	Major histocompatibility complex
*Mtb*	*M. tuberculosis*
PAMP	Pathogen-associated molecular pattern
PBMC	Peripheral blood mononuclear cell
pDC	Plasmacytoid dendritic cell
SNP	Single nucleotide polymorphism
TB	Tuberculosis
TLR	Toll-like receptor
VL	Viral load
