# Supplementary material for: Expression of Toll-like Receptors and Chemokine Receptors in the Early and Advanced Stages of HIV Infection Aggravated by Mycobacterium tuberculosis
Source: Int J Mol Sci. 2026 Sep 14;27(18):8161. doi: 10.3390/ijms27188161 (PMC13607093; doi:10.3390/ijms27188161)
Supplement: Supplementary file 1 [file ijms-27-08161-s001.zip › ijms-4463015-supplementary.pdf]

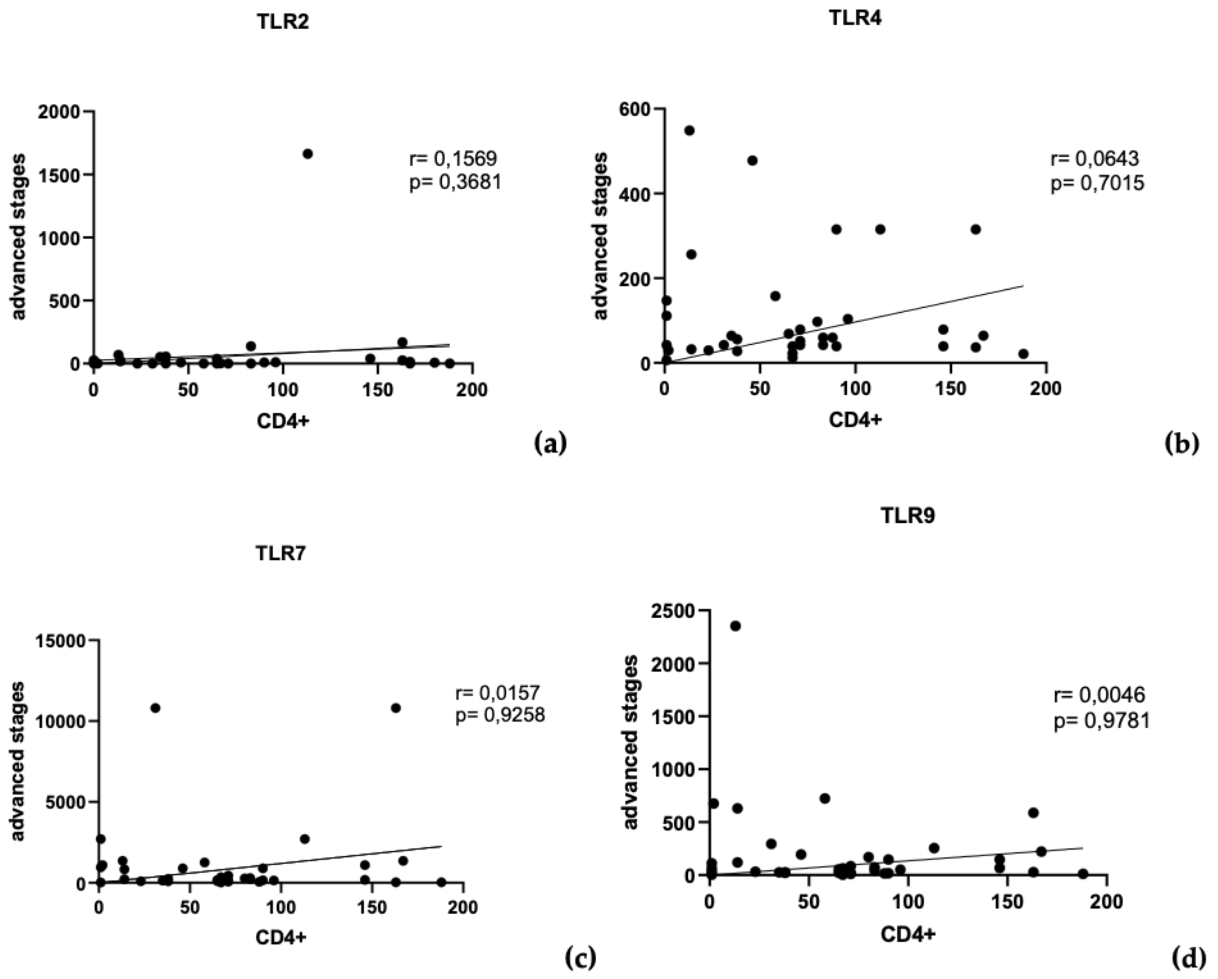

Supplementary Figure S1. Correlation between the expression of CD4+ cells and the expression of Toll-like receptors in HIV-infected patients with advanced stages of HIV infection

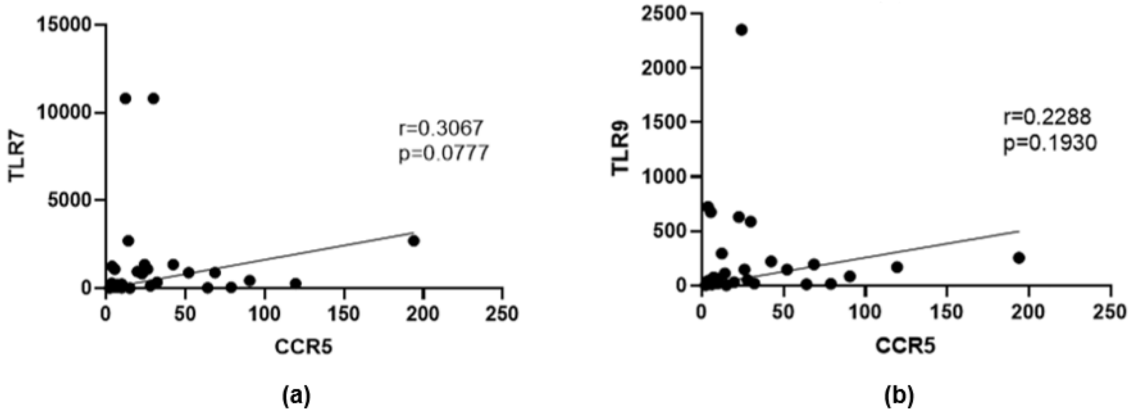

Supplementary Figure S2. Correlation between the gene expression level of CCR5 and the production of Toll-like receptors in HIV-infected patients with advanced stages of the disease: (**a**) CCR5/TLR7. (**b**) CCR5/TLR9. $2^{(-\Delta\Delta C(T))}$, normalized expression coefficient.

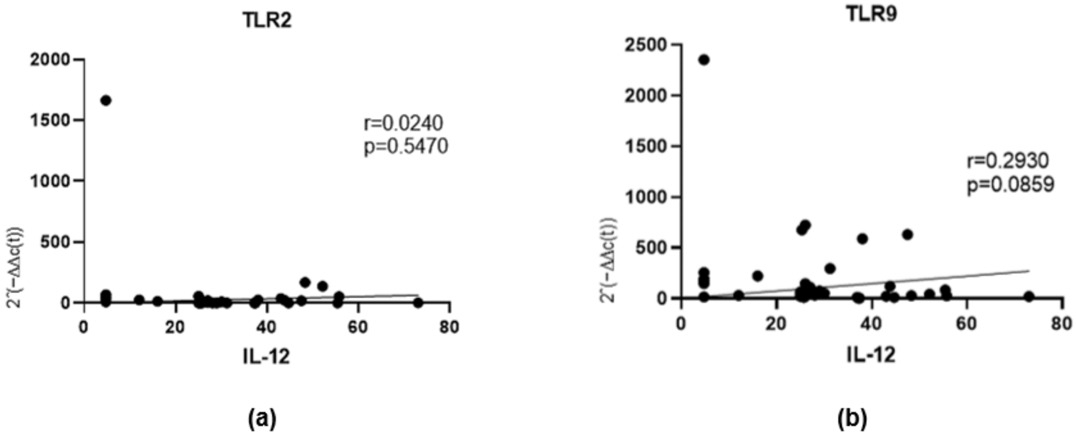

Supplementary Figure S3. Correlation between TLRs gene expression levels and IL-12 production in HIV-infected patients with advanced stages of HIV infection: (**a**) IL-12/TLR2. (**b**) IL-12/TLR9. $2^{(-\Delta\Delta C(T))}$, normalized expression coefficient.

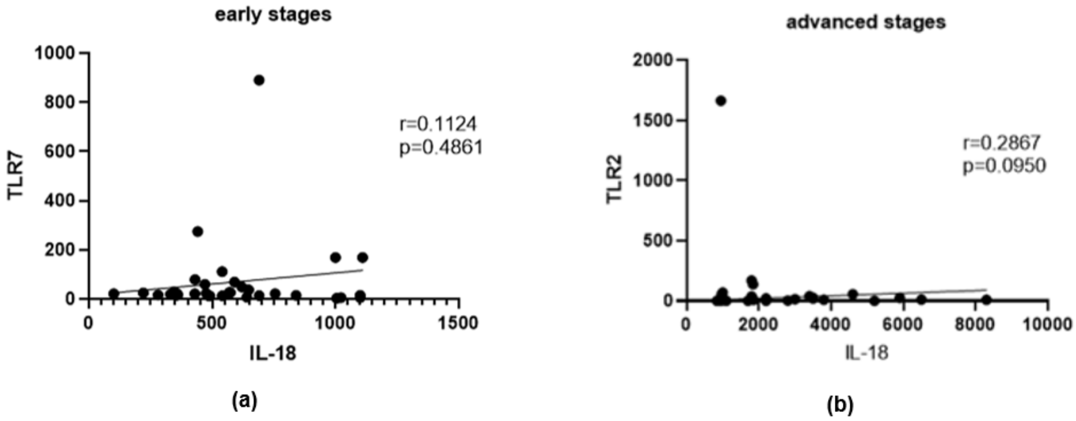

Supplementary Figure S4. Correlation between the gene expression levels of TLRs and the production of IL-18 in HIV-infected patients at different stages of HIV infection: (**a**) IL-18/TLR7 early stages. (**b**) IL-18/TLR2 advanced stages. $2^{(-\Delta\Delta C(T))}$, normalized expression coefficient.

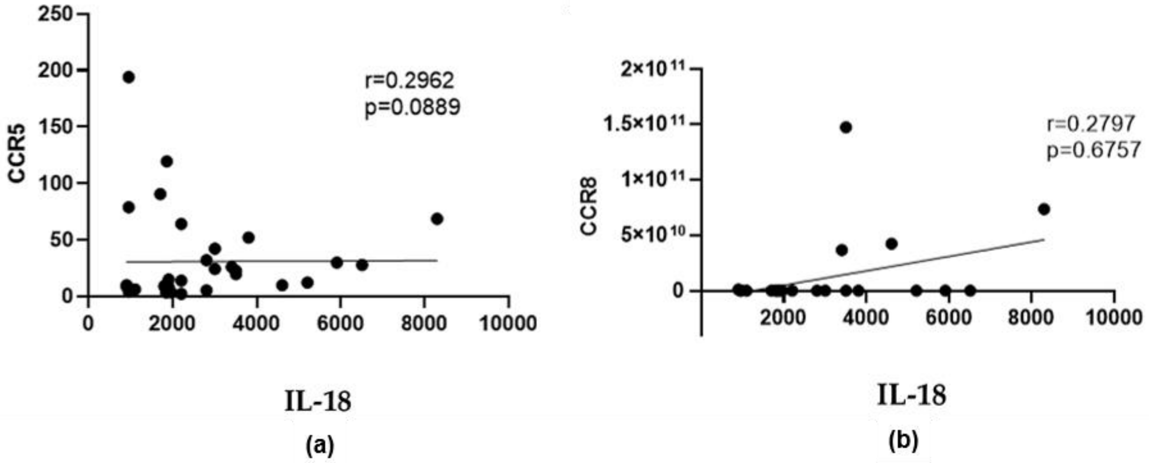

Supplementary Figure S5. Correlation between the gene expression levels of chemokine co-receptors and the production of IL-18 in HIV-infected patients at advanced stages of HIV infection: (**a**) CCR5. (**b**) CCR8. $2^{(-\Delta\Delta C(T))}$, normalized expression coefficient.
